# First United States Experience With Focal Pulsed‐Field Ablation Using the FARAPOINT Catheter for Refractory Ventricular Tachycardia

**DOI:** 10.1111/jce.70498

**Published:** 2026-09-13

**Authors:** Jorge L. Reyes, Rezwan Munshi, Collin Goebel, Venkat Tholakanahalli

**Affiliations:** ^1^ Cardiac Electrophysiology Section Minneapolis VA Health Care System and University of Minnesota Medical School Minneapolis Minnesota USA

**Keywords:** catheter ablation, FARAPOINT, focal ablation, pulsed‐field ablation, refractory, ventricular tachycardia

## Abstract

**Background:**

Drug‐refractory ventricular tachycardia (VT) after failed radiofrequency (RF) ablation remains a clinical challenge. Pulsed‐field ablation (PFA) is expanding to ventricular applications, but United States experience with focal pulsed‐field‐only catheters in VT is sparse, and pulsed‐field platforms are not equivalent across manufacturers. Here we describe the first United States experience of FARAPOINT focal PFA in patients with refractory VT.

**Methods and Results:**

We retrospectively reviewed ten consecutive patients who underwent VT ablation with the FARAPOINT (Boston Scientific) focal PFA catheter at a single VA Health Care System between July 2025 and May 2026. All patients had drug‐refractory sustained monomorphic VT. Median age was 77 years with a mix of ischemic and nonischemic cardiomyopathy. Six of ten had at least one prior failed catheter ablation. Acute non‐inducibility was achieved in all nine patients who underwent formal post‐ablation stimulation testing. No procedural complications occurred. Over a median follow‐up of 95.5 days, sustained VT recurred in four of ten patients. No patient underwent repeat ablation, required mechanical circulatory support, or died.

**Conclusions:**

In this initial United States experience, FARAPOINT was safe and acutely effective in patients with ischemic cardiomyopathy within a high‐risk cohort with refractory VT and prior failed ablations, including challenging substrates. Prospective multicenter studies are warranted to expand these observations.

## 1 Introduction

Drug‐refractory ventricular tachycardia (VT) after failed radiofrequency (RF) ablation remains a clinical challenge, owing largely to limited lesion depth in intramural and epicardial substrates. Pulsed‐field ablation (PFA) offers preferential myocardial targeting and tunable lesion depth and has expanded into ventricular applications [1, 2]. However, published ventricular experience remains limited, and PFA platforms are not equivalent across manufacturers. Catheter geometry, waveform, and ablation modality differ substantially, so platform‐specific data cannot be extrapolated. United States experience with focal pulsed‐field‐only catheters in VT is particularly sparse, with prospective data ongoing (FOCUS‐PFA; NCT06747013) [3]. We describe a United States case series of FARAPOINT (Boston Scientific, Marlborough, MA) focal pulsed‐field ablation in patients with refractory VT.

## 2 Materials and Methods

We retrospectively reviewed ten consecutive patients who underwent VT ablation with the FARAPOINT focal pulsed‐field catheter at the Minneapolis VA Health Care System between July 2025 and May 2026. Institutional review board approval and off‐label institutional authorization were obtained. Patients had drug‐refractory sustained monomorphic VT, and most had previously failed endocardial radiofrequency ablation. Procedures used intracardiac echocardiographic guidance, femoral access, and intravenous heparin (activated clotting time ≥ 350 s). Three‐dimensional electroanatomic mapping used the EnSite X system (Abbott, Plymouth, MN), with the HD Grid Mapping Catheter (Abbott). All pulsed‐field applications were delivered with the FARAPOINT catheter per the manufacturer's protocol; no concomitant radiofrequency ablation was performed. Acute success was defined as non‐inducibility of any sustained monomorphic VT (cycle length > 240 ms) after a standardized programmed stimulation protocol from the right ventricular apex and outflow tract (up to triple extrastimuli, with isoproterenol challenge). The primary clinical endpoint was sustained VT recurrence during follow‐up of at least 30 days post‐procedure.

## 3 Results

Ten consecutive male patients (median age 77 years [IQR 74 to 81]) underwent FARAPOINT‐based VT ablation (Table 1). Mean left ventricular ejection fraction was 34.2% ± 9.8%. Cardiomyopathy was ischemic in seven, nonischemic in two, and mixed in one. Six of ten patients had at least one prior failed catheter ablation (range 0 to 3), and no patient presented in VT storm. Eight patients had a prior implantable cardioverter‐defibrillator or cardiac resynchronization therapy defibrillator. Substrate distribution included basal inferior, inferolateral, or inferoseptal scar (five patients), apical scar (two), basal septal nonischemic substrate (one), and anterolateral papillary muscle (two). A median of 108 FARAPOINT applications were delivered per patient (IQR 21 to 142.) Acute non‐inducibility was achieved in all nine patients tested; in one patient, mapping‐confirmed loss of local capture was deemed sufficient, and stimulation testing was deferred. Remapping using high density multipolar catheter (Advisor HD grid, Abbott cardiovascular Inc. Plymouth MN) demonstrated elimination of late isochronal crowding, homogenization of farfield and near field electrograms and altered bipolar and unipolar voltages acutely. A representative case can be seen in Figure 1. No coronary vasospasm, phrenic nerve injury, stroke, pericardial effusion, vascular complication, or unanticipated ventricular fibrillation occurred, and capture‐threshold and sensing parameters were unchanged in patients with pre‐existing devices. Over a median follow‐up of 95.5 days (IQR 66 to 163; range 49 to 349), sustained VT recurred in four of ten patients at 23, 39, 48, and 326 days. One additional patient had non‐sustained VT on device interrogation, not categorized as sustained recurrence. Among the three patients with nonischemic or mixed cardiomyopathy, all experienced some form of ventricular arrhythmia recurrence during follow‐up, comprised of non‐sustained VT in one and sustained monomorphic VT in two. No patient underwent repeat ablation, required mechanical circulatory support, underwent transplantation, or died during follow‐up.

**Table 1:** Patient‐level characteristics and outcomes of the initial U.S. cohort treated with the FARAPOINT focal pulsed‐field ablation catheter for refractory ventricular tachycardia (*n* = 10). All patients were male.

Pt	Age	EF (%)	Etiology	Substrate	Prior abl. (*n*)	Acute non‐induc.	F/U (d)	VT recur.
1	75	28	ICM	Basal inferior, inferolateral, apical septal/lateral, basal septal	1	Yes	349	Yes (day 326)
2	78	33	ICM	Basal inferolateral, LV/RV basal septal, mid‐distal basal	2	Yes	177	No
3	57	25	NICM	Anterior mitral annulus, AMC, RV basal septal	3	Not tested^a^	163	NSVT only (day 80)
4	83	25	ICM + NICM	Basal inferior, inferolateral, inferoseptal	0	Yes	127	Yes (day 23)
5	73	38	ICM	Apical septal, apical inferior/inferolateral, mid inferoseptal/inferior	1	Yes	100	Yes (day 39)
6	72	58	NICM	Basal septum (with concomitant alcohol septal ablation)	3	Yes	83	Yes (day 48)
7	82	40	ICM	Anterolateral papillary muscle	0	Yes	91	No
8	78	28	ICM	Basal inferolateral, basal inferior	1	Yes	49	No
9	76	38	ICM	Basal inferior	0	Yes	66	No
10	82	33	ICM	Anterolateral papillary muscle	0	Yes	55	No

^a^ Patient 3 did not undergo formal post‐ablation programmed stimulation; mapping‐confirmed loss of local capture was felt sufficient by the operator.

**Figure 1:**
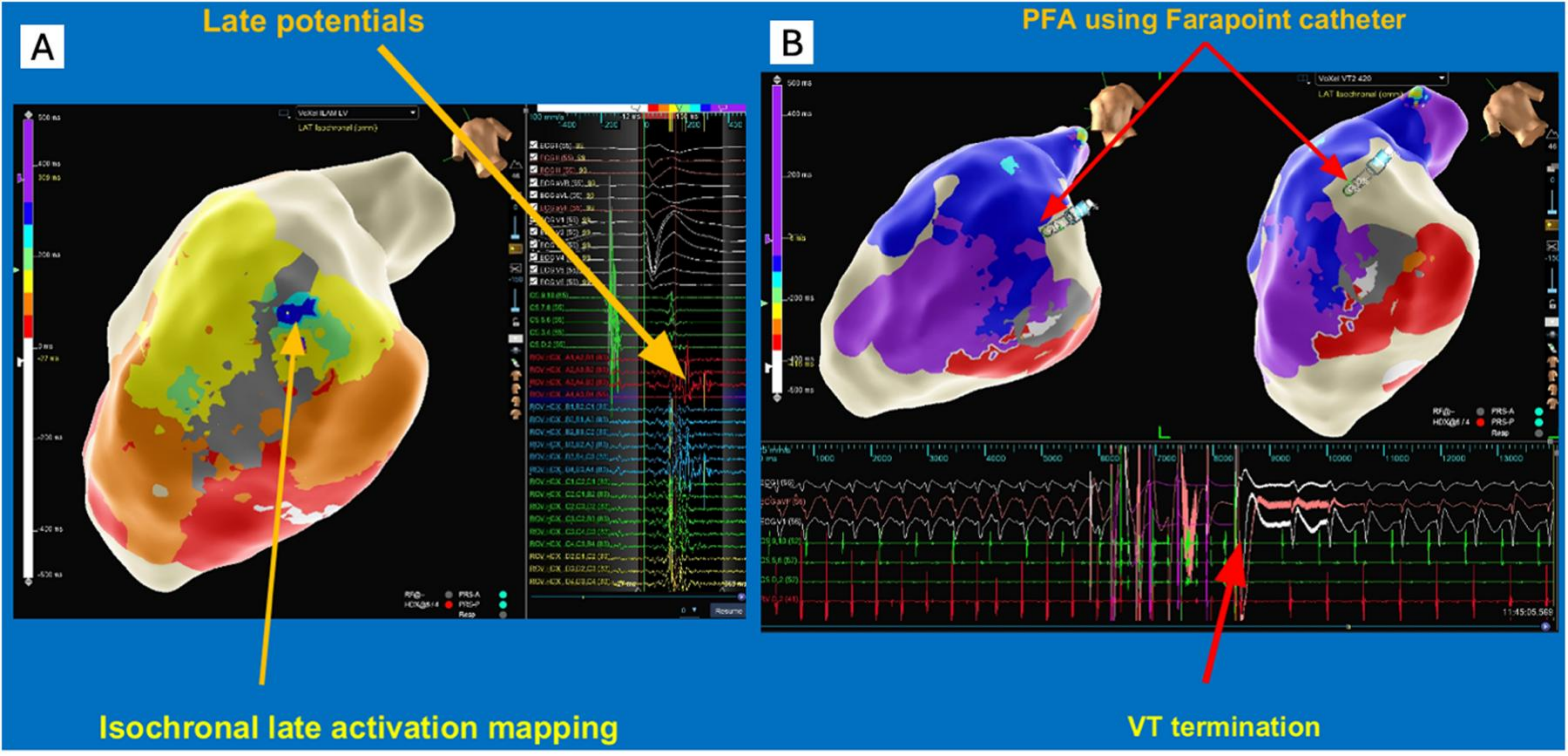
Representative case of focal pulsed‐field ablation (PFA) for refractory ventricular tachycardia (VT) using the FARAPOINT catheter (Boston Scientific, Marlborough, MA). A 78‐year‐old man with ischemic cardiomyopathy, a basal‐to‐mid inferior wall scar with dense calcification, and prior failed endocardial radiofrequency ablation of an entrainment‐confirmed clinical VT underwent re‐ablation. (A) Left ventricular endocardial substrate map during sinus rhythm, acquired with the EnSite X system and HD Grid Mapping Catheter (Abbott, Plymouth, MN). Voxel isochronal late activation mapping (ILAM) demonstrates crowding of isochrones within the basal‐to‐mid inferior wall scar, identifying a corridor of slow conduction. HD Grid electrograms recorded at the targeted site display discrete late potentials consistent with the arrhythmogenic substrate. (B) Activation map of the clinical VT (cycle length 420 ms) with the FARAPOINT catheter positioned at the critical isthmus identified in panel A. Surface electrocardiogram and intracardiac electrogram recordings during the first pulsed‐field application demonstrate abrupt termination of VT.

## 4 Discussion

In this initial U.S. experience with FARAPOINT for ventricular tachycardia, ablation was acutely effective and safe in a challenging cohort dominated by patients with recurrent VT after failed prior radiofrequency ablation. Our results demonstrated acute non‐inducibility in all nine tested patients and produced no procedure‐related complications. At follow‐up, patients with ischemic cardiomyopathy were free from recurrent VT in 71% of cases. However, in patients with non‐ischemic or mixed cardiomyopathy, two of three patients had recurrent sustained VT and the final patient had NSVT. Accordingly, our data do not support the efficacy of focal pulsed‐field ablation in nonischemic substrate. This observation, constrained by the small number of such patients, should be interpreted with caution. Notably, six of ten patients had previously failed endocardial RF ablation—a setting that often reflects substrate beyond the reach of conventional lesion depth, particularly intramural scar, deep septal substrate, and papillary muscle origins. PFA offers preferential myocardial targeting and tunable lesion depth without dependence on temperature or impedance feedback and may extend lesion penetration into substrate that defeated prior attempts. In this cohort, three of the six patients who had previously failed RF ablation were free from recurrent sustained VT at follow up. Importantly, no unanticipated ventricular fibrillation occurred, a complication recently described with large‐footprint dual‐energy platforms [4]. Capture‐threshold stability was preserved throughout, consistent with safety considerations highlighted by the cardioverter‐defibrillator failure in CLEAR‐VT [1] and the lead‐threshold rise in early dual‐energy ventricular series [5]. These observations support FARAPOINT as a small‐footprint, pulsed‐field‐only alternative to dual‐energy lattice‐tip platforms [6] for use in ischemic cardiomyopathy and extends focal pulsed‐field experience in outflow‐tract premature ventricular contractions to refractory sustained VT [2]. This study has several limitations. First, the cohort is small and from a single center, which precludes inferential statistics and limits generalizability. Second, follow‐up duration is relatively short, and longer‐term data are needed to establish durability. Third, one patient did not undergo formal post‐ablation programmed stimulation. Nonetheless, as the first United States experience with this catheter in refractory VT, this series is intended to demonstrate acute feasibility and safety to motivate prospective multicenter validation.

## 5 Conclusions

In this initial United States experience, FARAPOINT was found to be safe and effective in patients with ischemic cardiomyopathy complicated by refractory VT and prior failed ablations, particularly in substrates such as papillary, deep septal, and coronary venous targets where focal catheter–tissue contact and depth are preferred. Further research should be pursued to expand and confirm these observations and further evaluate efficacy in non‐ischemic cardiomyopathy.

## Funding

The authors have nothing to report.

## Ethics Statement

The study was conducted in accordance with the ethical principles of the Declaration of Helsinki and all applicable regulatory requirements. The study was reviewed and approved by the Institutional Review Board of Minneapolis VA Health Care. IRB approval 1594880; off‐label institutional authorization documented for all cases.

## Consent

Informed consent was obtained from all patients involved in this study.

## Conflicts of Interest

The authors declare no conflicts of interest.

## Data Availability

The data that support the findings of this study are available on request from the corresponding author. The data are not publicly available due to privacy or ethical restrictions.
